# Total parenteral nutrition-induced Wernicke’s encephalopathy after oncologic gastrointestinal surgery

**DOI:** 10.1515/med-2020-0210

**Published:** 2020-07-20

**Authors:** Piergiorgio Fedeli, Richard Justin Davies, Roberto Cirocchi, Georgi Popivanov, Paolo Bruzzone, Michela Giustozzi

**Affiliations:** School of Law - Legal Medicine, University of Camerino, Camerino, Italy; Department of General Surgery, Cambridge Colorectal Unit, Addenbrooke’s Hospital, Cambridge University Hospitals NHS Trust, Cambridge, United Kingdom; Department of Surgical Science, University of Perugia, Perugia, Italy; Department of Surgery, Military Medical Academy, Sofia, Bulgaria; Dipartimento di Chirurgia Generale e Specialistica “Paride Stefanini”, Sapienza Università di Roma, Rome, Italy; Department of Medicine, Internal Vascular and Emergency Medicine and Stroke Unit, University of Perugia, Perugia, Italy

**Keywords:** Wernicke’s encephalopathy, parenteral nutrition, malpractice

## Abstract

Carl Wernicke described the disease bearing his name in 1881 and reported three cases characterized by the presence of mental confusion, ataxia, and ophthalmoplegia. Wernicke’s disease is mainly observed in alcoholic patients, due to decreased vitamin intake as a consequence of an unbalanced diet, and a reduction of absorption due to the effects of alcohol. Likewise, inadequate vitamin intake is prevalent in older patients. Wernicke’s encephalopathy due to inappropriate total parenteral nutrition (TPN) occurs infrequently; recently, there is an increase in the literature concerning Wernicke’s encephalopathy in patients after general and bariatric surgeries. We present two cases of Wernicke’s encephalopathy after oncologic gastrointestinal surgery by failure to administer vitamin B1 during TPN; to our knowledge, these are the first two cases of Wernicke’s encephalopathy after colorectal surgery for cancer. In our opinion, timely diagnosis and treatment are mandatory to avoid nonfunctional recovery and consequent malpractice legal actions as well as an increase in the health-care costs correlated with the prolonged hospital stay and with the nonfunctional recovery.

## 1 Introduction

Carl Wernicke described the disease that bears his name in 1881, reporting three cases characterized by the presence of mental confusion, ataxia, and ophthalmoplegia; one of these patients had pyloric stenosis due to ingestion of caustics, and the other two were alcoholics [1]. Wernicke could not attribute this disease to vitamin deficiency (vitamin B1), because at that time, vitamins were still unknown. In 1912, Casimir Funk isolated elements in raw white rice and called them for the first time “vitamins” because they were considered indispensable for life [2]. In 1926, Jansen and Donath isolated pure thiamine. In 1929, the Nobel Prize for medicine was awarded to Christian Eijkman and Frederick Gowland Hopkins for having identified that beriberi was linked to the nutritional deficiency of vitamins [3].

Numerous efforts were made to study beriberi, leading to the definition of the role of thiamine deficiency in its aetiology and consequently of its fundamental importance as a therapy.

Vitamin B1 is a coenzyme essential for the integrity of membranes and, therefore, for the normal function of the nervous system, muscles, and heart. Due to its average life of approximately 2 weeks in humans, there are no reserves, and therefore, in people who do not take it with food, deficiency symptoms occur after around 3 weeks [4,5].

The clinical manifestations in critically ill patients are nystagmus and ophthalmoplegia, cerebellar dysfunction (ataxia), confusion, and altered mental status.

Wernicke’s disease incidence is higher in alcoholics, both as a consequence of the decrease of vitamin intake from a very unbalanced diet and due to reduced absorption in alcoholic patients. Data suggest that 3–12% of alcoholics admitted in hospital have clinical signs of Wernicke’s encephalopathy [6,7,8]. It may also affect patients with malabsorption syndrome, leading to chronic diarrhoea.

Likewise, inadequate vitamin intake is prevalent in older adults. Some studies have shown a higher risk of deficiencies in senior citizens living alone or in a psychiatric or nursing home environment [9,10,11,12].

Wernicke’s encephalopathy in hospitalized patients can be caused by two factors: insufficient thiamine content within total parenteral nutrition (TPN) preparations [13,14] or an increase in the requirement if perfusions of glucose solutions used, as the metabolism of glucose consumes thiamine [15,16,17].

In both cases, Wernicke’s encephalopathy should be considered an iatrogenic complication [18,19].

We report two cases of Wernicke’s encephalopathy that could have been harbingers of malpractice legal actions.

## 2 1th Case report

A 49-year-old male without alcohol addiction underwent subtotal gastrectomy with D2 lymphadenectomy for a locally advanced antral cancer (T3N1M0) and was discharged on the seventh postoperative day. The same evening, while at home, the patient developed sudden diffuse abdominal pain. On next morning, he was readmitted to the hospital, and emergency surgery was performed for peritonitis from dehiscence of the duodenal stump. The patient was transferred to the intensive care unit, where TPN was started. During 2 weeks of TPN, vitamin B1 was not administered. On the 20th postoperative day, there was a gradual appearance of neurological symptomatology and a neurologist arranged transfer to the neurological unit. Ophthalmological examination suggested a partial horizontal and vertical ophthalmoplegia with prominent gait ataxia; a brain computed tomography ruled out organic diseases. The patient was later transferred to another hospital with the diagnosis of “Antral gastric cancer (T3N1M0): subtotal gastrectomy. Acute peritonitis by late duodenal perforation. Septic state. Central nervous disorders of likely toxic origin.” In this hospital, in addition to strict surgical control of the patient’s condition, parenteral nutrition (later on replaced by enteral nutrition) with high-dose parenteral thiamine (500 mg intravenously every 8 h) was immediately started; neurological symptoms slowly improved after 5 days; successively, the dose of thiamine was reduced (100 mg intravenously every day). The patient was discharged after 30 days with the diagnosis of “Outcomes of subtotal gastrectomy with post-operative course complicated by duodenal dehiscence. External biliary fistula. Dizzy syndrome.” At discharge, the surgeons recommended a neurological check-up and therapy with thiamine (100 mg/day orally). He was then admitted to an academic neurology department for another clinical evaluation; the final diagnosis was “Wernicke’s vitamin deficient encephalopathy.” One year later, the patient presented with a cerebellar syndrome, which substantially prevented him from performing movements that required good coordination. This clinical picture made many acts of daily life difficult, such as feeding and personal hygiene, with walking severely limited.

## 3 2nd Case report

A 59-year-old female with previous *mixed dyslipidemia and hypothyroidism* underwent laparoscopic anterior resection of the upper rectum for cancer. On the third postoperative day, she complained of continuous vomiting, so the surgeons suspected a small bowel obstruction due to postoperative adhesions. The patient underwent non-operative management (NOM); a central venous catheter was placed, and TPN was started, administering Clinimix^®^ (amino acids and glucose solution). Vomiting persisted, and a mild acute pancreatitis was also diagnosed. The patient was then transferred to the Gastroenterology Unit where the Clinimix^®^ was replaced with Nutriplus Lipid^®^ (including lipid infusion), but no vitamins were administered. Subsequently, the patient did not respond to verbal stimuli; the consultant neurologist described her as “vigilant, oriented, collaborative patient, no segmental force deficits to the limbs. Symmetrical osteotendinous reflexes have been observed. Nystagmus is present with coarse shocks in the extreme lateral gaze” and transferred the patient to the Neurology Unit, suspecting myelinolysis. She was given a vitamin complex (Cernevit^®^) with high-dose parenteral thiamine (500 mg intravenously every 8 h). The duration of high-dose therapy was 3 days; successively, the patient received a standard dose of 100 mg thiamine daily. Enteral nutrition was later started and continued, with a progressive improvement in the mental state, ophthalmoplegia, and the ability to eat normally. Due to neurological disease and prolonged hospital stay, she was transferred to a Rehabilitation Centre for a further 4 months, obtaining only a moderate clinical improvement. One year later, eyelid ptosis, pyramidal hyperreflexia in the four limbs, improved balance, and persisting dysphasia, dysarthria, and dysphagia were observed.


**Consent for publication:** Written informed consent was obtained from the patients for publication of these two case reports.

## 4 Discussion

Wernicke’s encephalopathy due to inappropriate TPN occurs infrequently [20,21,22,23]. However, an increasing number of papers have been published [24,25,26,27,28], reporting this disease after general and bariatric surgeries and thereby raising the possibility of malpractice legal actions [24,25,26,27,28,29].

In 2008, Aasheim reported the results of a systematic review of the literature on this topic, including 84 patients with Wernicke’s encephalopathy after bariatric surgery (gastric bypass or a restrictive procedure had been performed in 80 cases [95%]). From 2008 until 2020, 224 new publications described this disease.

Our two cases had postoperative complications and prolonged TPN was needed. In both patients, after more than 3 weeks from the beginning of the TPN, signs and symptoms related to Wernicke’s encephalopathy were observed. The cause was identified as absolute deficiency of vitamin B1.

This diagnosis was delayed in both patients; late high-dose parenteral thiamine administration reversed some of the acute effects of Wernicke’s encephalopathy. These delays reduced the effectiveness of the treatment, limiting it to improvement without returning to normal clinical conditions.

In everyday clinical practice, traditional regimens include 100 mg of parental (intravenous or intramuscular) thiamine administration for 3–7 days (treatment period), followed by oral thiamine indefinitely. In these two cases, the neurological condition of patients was more severe, and therefore, higher doses (up to 1 g of intravenous thiamine daily) were needed to obtain clinical improvement. An accurate selection of patients is very important; the antioxidant effect of vitamin B1 may change in oncological patients receiving cytotoxic drugs [30]. This hypothesis was supported from a recent study performed by Restivo and colleagues. The authors evaluated 45 patients who underwent gastrointestinal surgery due to cancer (2 total gastrectomies, 4 partial gastrectomies, 9 right hemicolectomies, 2 left hemicolectomies, 17 anterior resections of rectum, 3 abdominal perineal resections, and 4 other colonic resections). A diagnosis of Wernicke was reported in 4.4% at discharge. The authors suggested that “cancer patients submitted to gastrointestinal surgery should be considered at risk of Wernicke and should receive thiamine prophylactic treatment” [31].

The accurate study of medical records, with day-to-day control of components used for TPN, allowed us to highlight these effects of the lack of vitamin B1 in the solutions used for intravenous feeding. This complication should have been avoided given that clinical experience of TPN in surgical patients dates back more than 40 years. In the literature, very few cases of Wernicke’s encephalopathy were reported after oncologic gastrointestinal surgery during TPN, the localization of gastrointestinal cancer was the stomach and the pancreas (Table 1) [32,33,34,35,36,37,38,39,40,41,42,43,44].

**Table 1:** Review of literature: case report of Wernicke’s encephalopathy after oncologic gastrointestinal surgery

Author and year	Type of article	Location of cancer	Type of surgery
Kim 2019	Case report	Pancreas	Pancreaticoduodenectomy
Tozzo 2017	Case report	Gastric	Gastrectomy
Tsao 2017	Case report	Gastric	Gastrectomy
Kilinc 2015	Case report	Gastric	Gastrectomy
Busani 2014	Case report	Pancreas	Pancreaticoduodenectomy
Karayiannakis 2011	Case report	Pancreas	Pancreaticoduodenectomy
Onieva-González 2011	Case report	Pancreas	Pancreaticoduodenectomy
Attard 2006	Case report	Gastric	Gastrectomy
Iwase 2002	Case report	Gastric	Gastrectomy
Arai 1997	Case report	Gastric	Gastrectomy
Batori 1995	Case report	Gastric	Gastrectomy
Shimomura 1998	Case report	Gastric	Gastrectomy

In a 1979 publication, the authors stated that “the intake of vitamins consists of vitamins C, K, complex B, folic acid.” In those years, the attending physicians or the hospital pharmacy independently provided TPN solutions [45]. Later on several pre-packaged industrial products have been introduced in clinical practice, substantially improving safety and quality. TPN industrial bags contain nutritional macroelements and electrolytes but still require a supplement of vitamins and trace elements before their infusion.

The physician who prescribes the administration of two- or three-compartment industrial TPN bags must indicate the required additive components; vitamins must be added just before use or they should be administered in a different solution.

Physicians who take care of patients needing TPN, as well as involved dieticians and pharmacists, must be aware of this clinical information [46].

Information for the prevention of Wernicke’s encephalopathy in patients undergoing digestive surgery and particularly gastric procedures has been published [47,48,49]. The guidelines of the European Federation of Neurological Societies for diagnosis, treatment, and prevention of Wernicke’s encephalopathy [50] provide guidance for monitoring surgical patients, mainly after the bariatric surgery [51,52,53,54,55].

## 5 Conclusion

In our two case reports, the following same errors occurred: late diagnosis, made by different clinicians, of Wernicke’s encephalopathy after use of TPN to manage complications of oncologic digestive surgery. Failure to administer vitamin B1 was the cause in these cases of encephalopathy. This complication led to serious implications for the patients and to an increase in healthcare costs due to prolonged hospital stay and incomplete recovery.
